# Interleukins 10 and 1β gene polymorphisms in ischemic stroke risk in the Egyptian population

**DOI:** 10.1038/s41598-025-98531-w

**Published:** 2025-05-14

**Authors:** Omali Y. El-Khawaga, Afaf M. ElSaid, Wessam Mustafa, A. N. El-Daw, Mariam Saad

**Affiliations:** 1 Biochemistry Division, Chemistry Department, Faculty of Science, Mansoura University, Mansoura, 35516 Egypt; 2 Genetic Unit, Department of Pediatrics, Faculty of Medicine, Mansoura University, Mansoura, 35516 Egypt; 3 Neurology Department, Mansoura University Hospital, Mansoura, 35516 Egypt

**Keywords:** Genetic mutation, Stroke, Cytokines, ARMS-PCR, IL-10, IL-1β, Biochemistry, Biological techniques, Cancer

## Abstract

Stroke remains the leading cause for lasting disability and death globally. Two frequent proinflammatory cytokine variants in the IL-10 and IL-1β genes, rs16944 T/C and rs1800896 G/A, may be major candidate gene loci impacting ischemic stroke vulnerability. The present case–control research aims to establish a link between both of these polymorphisms and ischemic stroke risk in the Egyptian population. The study demonstrates that the TC genotype, over dominant (TT + CC vs. TC) and dominant models (TC + CC vs. TT), exhibited greater prevalence among stroke groups as compared to the healthy group. Regarding IL-10, GA genotype, A allele, over dominant (GG + AA vs. GA), and dominant (GG vs. GA + AA) genotyping models in patients, they show highly significant differences from controls that increased the risk of stroke. Stroke patients with higher cholesterol, LDL, SBP, DBP, and lower HDL levels were more likely to develop stroke. The study found no significant link between genetic polymorphisms and smoking, gender, diabetes, or hypertension in stroke patients, except for the IL-1β heterozygous TC and dominant model, which was associated with troubles in chewing and swallowing and consciousness issues in the patients. Only troubles in chewing and swallowing manifestations were associated with the IL-10 variant. The rs16944 and rs1800896 polymorphisms differ substantially between groups, providing a more definite outcome for the Egyptian population and IS. The results validate the utilization of the rs16944 T/C and rs1800896 G/A variations in stroke prediction for Egyptians.

## Introduction

Stroke represents the world’s second-biggest cause of fatality^1^, with ischemic stroke (IS) incorporating 87% of stroke events. This cerebrovascular event, which is characterized by ischemia or necrosis, imposes major emotional and financial costs on afflicted people and society, resulting in a decrease in patient quality of life in developed as well as developing nations^2^. With a rise in the older population, the number of cases of IS is anticipated to be high (963/100.000 individuals), and strokes occur approximately 150.000:210.000 times each year. According to official national data, circulatory system disorders, including stroke, are the leading causes of mortality in Egypt, in which stroke represents 6.4% of all fatalities and ranks third behind cardiac and gastrointestinal conditions. Even though the overall rate of stroke deaths has decreased in many nations, it has remained relatively stable in Egypt over the last decade^3^.

IS etiology and pathogenesis research has been serious, yet the fundamental mechanism remains unknown. As a complicated illness with various risk factors, including cerebral atherosclerosis or subsequent ischemic brain damage, which can lead to cerebral thrombosis and IS. Other factors to consider include hypertension, pollution from the environment, genetic predisposition, and unhealthy lifestyle habits. There is growing evidence that a substantial polygenic inheritance component for IS etiology exists, including aberrant patterns of gene expression as well as genetic alterations^4^. Inflammatory compounds, as well as SNPs in genes expressing inflammatory cytokines, contribute to the onset and progression of a wide range of clinical illnesses, including cardiovascular disease^5,6^.

Interleukins (ILs) are powerful inflammatory regulators. Excessive inflammation is widely known to be an etiological factor in atherosclerotic and thrombotic vascular disorders, including IS^7,8^. As a consequence, functional interleukin variations, which may modify interleukin function, may also influence individual vulnerability to IS. IL-1β, a proinflammatory cytokine, promotes the release of chemokines implicated in inflammation and can be affected by genetic differences. It promotes arterial injury and atherosclerosis by increasing the growth and differentiation of cells as well as the production of matrix-degrading enzymes^9^.The rs16944 T/C mutation in the promoter area of the IL-1β gene affects the production of IL-1β^10^. This SNP can influence transcriptional activity, with the T allele generally associated with more IL-1β production than the C allele. Elevated IL-1β levels caused by the T allele have been related to greater vulnerability to inflammatory illnesses, including rheumatoid arthritis, Crohn’s disease, and psoriasis, as well as mental health issues like schizophrenia. Mutations are additionally linked to the progression of cancer, cardiovascular disease, neurological illnesses, and metabolic problems such as diabetes^11^. IL-10 represents a multipurpose anti-inflammatory cytokine, mainly released by monocytes and lymphocytes. A temporary rise in IL-10 levels in plasma, cerebrospinal fluid, or blood mononuclear cells was identified in individuals suffering from acute stroke^12^. The IL10 rs1800896 (A/G) mutation in the promoter area of the IL10 gene affects the synthesis of interleukin-10 (IL-10), an important anti-inflammatory cytokine. This single nucleotide polymorphism changes guanine (G) to adenine (A), which can change the activity of transcription and the amount of IL-10 that is expressed. The G gene is often linked to higher levels of IL-10 production, which may lower inflammation but make it harder for pathogens to get rid of cells. On the other hand, the A allele may lower IL-10 levels, making people more likely to get inflammatory diseases. This polymorphism has been associated with several diseases, notably autoimmune conditions, infections, and cancer^13,14^. Earlier studies pointed to the possible link of IL-10 and IL-1β polymorphisms with IS and associated conditions^9,15^. However, the distribution of SNP spots in the aforementioned interleukins varies among racial and ethnic groups.

To the author’s best knowledge, there has been no publication assessing the consequences of the preceding IL-1β and IL-10 variation by case–control approach in the Egyptian population, which is more desirable than a cross-sectional study in terms of hazards evaluation. Here, to assess the significance of those mutations on the risk of stroke in consideration of confounding factors such as hypertension, smoking status, diabetes mellitus, and sex, we conducted this comparative study on the potential relationship between the rs16944 T/C mutation in the IL-1β gene and the rs1800896 G/A in the IL-10 gene and the probabilities of ischemic stroke in the Egyptian population.

## Materials and methods

The department of cardiology within Mansoura University Hospital, Egypt, was the site of this research work from June 2022 to April 2023. Following approval by the hospital’s ethical council, 100 verified IS patients as well as 150 healthy volunteers were chosen for this research study. Informed consent was obtained from all the subjects included in the study, and the study was approved by the Ethical Committee of the Faculty of Medicine, Mansoura University (IRB: MS.22.05.2009, date: 31/5/2022). All experiment procedures, including specimen collection, biochemical analysis, DNA isolation, and genotyping, have been performed according to relevant guidelines and regulations.

All the patients were examined by a qualified stroke neurologist, and ischemic stroke was differentiated by CT scans and MRI. Nobody in the healthy group smokes, has a history of a complicating disease, or uses drugs on a regular basis. The research excluded patients with significant cardiac, tumour, renal, and hepatic diseases, as well as thyroid or rheumatologic disease and a history of immunosuppressive or analgesic therapies from the study^16^. A systematic questionnaire was used to obtain information on demography and risk variables. All individuals got a thorough physical examination, as well as a regular biochemical blood lipid profile. Patients reported hypertension, diabetes, conscious status, swallowing issues, and smoking status. The controls showed no clinical or radiological indications of cerebrovascular illness.

### Biochemical analysis

Following an 8-h fast, specimens of blood were collected in the early hours of the day. A portion of the blood sample was put in glass tubes, allowed to clot at room temperature, and then applied to quantify serum lipid levels. Enzymatic procedures were employed to assess the quantities of TG, TC, LDL-C, and HDL-C in samples utilizing available commercial kits: TG (BioMed-(#TG117090) Triglycerides L.S., Cairo, Egypt); TC (BioMed-(#CHO104090) Cholesterol-LS, Egypt); and HDL-C (BioMed-HDL (#HDL114100).

### DNA isolation and genotyping of IL-10 and IL-1β polymorphisms

A total of five ml of blood was taken in EDTA tubes from all participants. Leukocytes were isolated from samples, and genetic material was subsequently extracted using the common techniques of a commercial Easy DNA Purification Pure^®^ kit (Cat. No. EE121-01, Transe GEN Easy Pure^®^). DNA was measured via UV light absorption spectrometry at a 260 nm wavelength. A contaminant-free specimen was defined as one with a 260/280 ratio between 1.7and 2.0. Samples were adjusted approximately 25 ng/µL for genotyping.

The samples were subsequently analyzed using the (ARMS)—Amplified Refractory Mutations System—PCR technique, followed by electrophoresis of gel. The primers for the reaction were designed previously to amplify genomic DNA fragments containing the SNP at locations − 1087 (G/A) in the promoter region of the human IL-10 gene sequence (GenBank: X78437.2)^17^ and IL-1β -511 (C/T) gene sequence (AJ224149)^18^ Table 1 lists all primer sequences utilized. For IL-1β genotyping, two tubes were utilized for the determination of the variant. Each PCR mix for the reaction had an overall amount of 32 µl and included 8 μl of external primers (4 μl of reverse control (RO) and 4 μl of forwarding control (FO)), 4 μl of DNA, and 4 μl of C allele primer for tube 1 and 4 μl of T allele primer for tube 2 combined with 16 μl of master mix (COSMO PCR RED Master Mix (W10203001), willow fort). For IL-10 gene (rs1800896) polymorphisms, each PCR reaction mixture was carried out in a total volume of 30 µl containing 6 µl of external primers (3 µl of FO and 3 µl of RO), 15 µl of master mix, and 3 µl of DNA in a thin-walled PCR tube. 3 µl of F (A) and 3 µl of R (G) primers were added^17^. The PCR samples were run through an Eppendorf Gradient Thermo cycler before being amplified on a T professional thermocycler (Biometra, Germany).

**Table 1:** Primer sequences used for IL-1β (rs16944) and IL-10 gene (rs1800896) polymorphisms^17,18^.

Mutation	Primer sequence	Size (bp)
IL-1β (rs16944) C/T	R (C): 5ʹ-CCTGCAATTGACAGAGAGCTAC C-3ʹ	220
	F (FO): ATCTGGCATTGATCTGGTTCATCC-3ʹ R(RO): 5ʹ-CTTAACTTTAGGAATCTTCCCACTT-3ʹ	
		150
	F (T): 5ʹ-CTTGGGTGCTGTTCTCTGCCGCA-3ʹ	
IL-10 gene (rs1800896) G/A	Common F (FO): 5¢-CCAGTTACAGTCTAAACTGGAATGCAG	430
	Common R(RO): 5¢-CTTGGATTAAATTGGCCTTAGAGTTTCT	
	R (G allele): 5¢-ACTTTCCTCTTACCTATCCCTACTTCACC	288
	F (A allele): 5¢-AACACTACTAAGGCTTCTTTGGGCAA	197

PCR was performed using the following temperature profile: (95 °C for 2 min) for the initial denaturation step, followed by thirty cycles of (95 °C for 30 s) for the denaturation, (60 °C for 20 s) for annealing, (72 °C for 30 s) for the extension step, and (72 °C for 5 min) for final extension for the rs16944. For rs1800896, the initial denaturation step was performed at 94 °C for 2 min, followed by thirty-five cycles of denaturation at 94 °C for one min, annealing at 62 °C for one min, the extension step at 72 °C for one min, then the final extension at 72 °C for two min. The byproducts of PCR have been separated on an agarose gel (2.5%), which was stained with ethidium bromide and then visualized by UV transillumination.

### Statistical analysis

The statistical evaluation was evaluated using SPSS version 25 (2017, Armonk, NY: IBM Corp.). The equilibrium of Hardy–Weinberg was determined employing the chi-square test. Data for continuous variables were reported as medians (ranges), while categorical variables were expressed as percentages of the total. The effects of genotypes were analyzed and presented for all three genotype groups. The P value was obtained after adjusting for covariates. The estimation of the connection between the IL-1β/10 profiles and stroke was evaluated by Fisher’s exact test. An odds ratio (OR) comprising a 95% confidence interval was then calculated. Statistical tests were deemed significant if the P-value was < 0.05^19^.

## Results

Table 2 lists the fundamental and clinical aspects of the population under investigation. The median age was 64 years for both patients and healthy controls. There is no significant difference in age or gender (P = 0.839 and P = 0.253, respectively). Risk factors for stroke showed a statistically significant association with blood pressure (DBP/SBP) (P < 0.001 for both). Other biochemical risk markers, including the levels of TC and LDL-C, rose in the level from 162.2 to 211 (P = 0.02), and from 103 to 140 (P < 0.001), respectively. On the other hand, the HDL-C level and the TG level showed a lower significant change from 30.23 to 35.6 (P < 0.001) and from 173 to 119 (P < 0.001), compared to healthy volunteers, respectively.

**Table 2:** Clinical characteristics of stroke patients and healthy controls.

		Stroke patients n = 100	Control n = 150	Test (p)	*P*
Age in years		64 (19–88)	64 (20–85)	T = 0.204	0.839
Gender (male/female)		60/40	79/71	X2 = 1.307	0.253
SBP (mmHg)		132 ± 19.04	118 ± 5.0	t = 8.51	< 0.001*
DBP (mmHg)		83 ± 11.32	74 ± 6.0	t = 8.17	< 0.001*
TG (mg/dl)	(range) median	(58–378) 108.5	(67–167.7)120	U = 9591	< 0.001*
TC (mg/dl)	(range) median	(105–437) 211	(100–198)162.2	U = 11,741	0.001*
HDL-C (mg/dl)	Mean ± SD	35.55 ± 5.76	50 ± 6.0	T = 18.9	< 0.001*
LDL-C (mg/dl)	(range) median	(80–180) 140	(79–134)103	U = 2509.0	< 0.001*

Significant values are in bold.

*P* probability, *χ*^*2*^ Chi-square test, *U* Mann–Whitney, *T* Student t-test, *significant.

### Genetic model, genotyping, and allelic frequency of IL-1β (rs16944) and IL-10 rs1800896 gene polymorphism of stroke patients compared to healthy controls

**Fig. 1:**
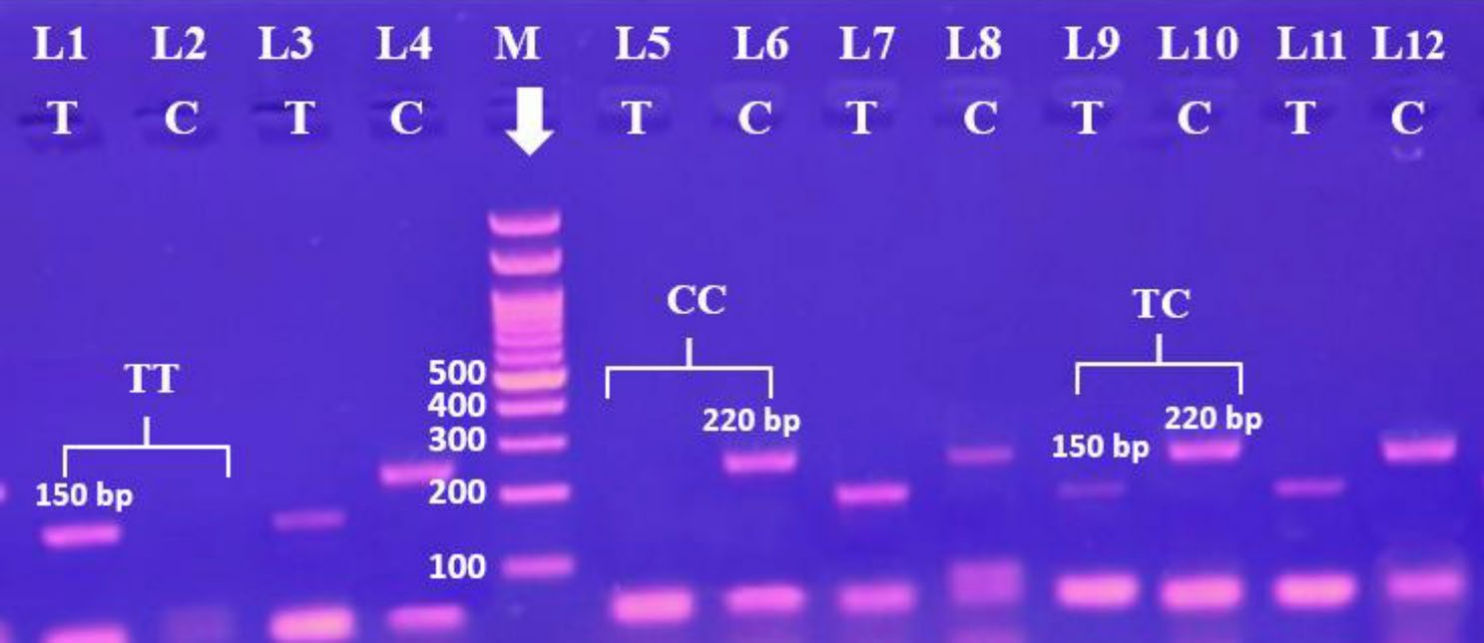
Gel electrophoresis of PCR product of IL-1β rs16944 by ARMS PCR; where each two lanes represent single participant. M indicates for 100 bp DNA marker. 150-bp bands indicate the T allele, whereas particular 220-bp bands indicate the C allele. Lanes (1and 2) indicate TT homozygous genotype; whereas the T allele appears in lane 1 and the C allele absents from lane 2. Lanes (3, 4, 7, 8, 9, 10, 11 and 12) indicate TC heterozygous; where the T allele appears at lanes (3, 7, 9, and 11) at 150 bp and the C allele appears at lanes (4, 8, 10, and12) at 220 bp. Lanes (5 and 6) are CC homozygous, where the C allele appears at lane 6 at 220 bp and the T allele absents from lane 5.

Applying the Hardy–Weinberg equation and calculating the expected count indicated that all of the examined SNP genotypes in both the control and case groups were in HW equilibrium. Table 3 demonstrates a highly significant statistical difference in IL-10 and IL-1β genotype polymorphisms from controls. The homozygous genotype TT was observed in 11% of stroke patients and in 22% of healthy controls. Patients had a lower CC homozygous genotype (7%) than controls (20.7%) but a higher TC heterozygous genotype (82%), compared to the control group (57.3%). The TC genotype (odds ratio [OR] 2.860; probability (P) = 0.006), (TT + CC Vs TC) over dominant (OR 3.390; P =  < 0.001), and (TC + CC Vs TT) dominant models (OR 2.282; P = 0.028) showed higher frequency among the stroke group when compared to the healthy group, with a high risk to develop stroke, while the (TC + TT Vs CC) recessive model showed lower frequency among the stroke group when compared to the control group with a protective effect against stroke (OR 0.289; P = 0.005). There were no notable variations in the CC genotype or C allele between controls and patients (p > 0.05 for both) (Fig. 1).

**Table 3:** IL-1β (rs16944) and IL-10 (rs180089) gene genotypes and alleles frequency between healthy controls and stroke patients.

			Control (150)	Stroke (100)	*P*	OR (95% CI)
			n (%)	n (%)		
IL-1β rs16944 polymorphism	General	TT	33 (22.0)	11 (11.0)	**–**	Reference
		TC	86 (57.3)	82 (82.0)	0.006	2.860 (1.356–6.034)
		CC	31 (20.7)	7 (7.0)	0.474	0.677 (0.233–1.969)
	Recessive	TT + TC Vs CC	119 (79.3)	93 (93)	**–**	Reference
			31 (20.7)	7 (7.0)	0.005	0.289 (0.122–0.685)
	Over-dominant	TT + CC Vs TC	64 (42.7)	18 (18)	**–**	Reference
			86 (57.3)	82 (82.0)	< 0.001	3.390 (1.853–6.203)
	Dominant	TT Vs TC + CC	33 (22.0)	11 (11.0)	**–**	Reference
			117(78)	89 (89)	0.028	2.282 (1.093–4.764)
	Allelic	T-Allele	152 (50.7)	104 (52.0)	**–**	Reference
		C-Allele	148 (49.3)	96 (48.0)	0.770	0.948 (0.663–1.356)
IL-10 rs180089 polymorphism	General	GG	71 (47.3)	7 (7.0)	–	Reference
		GA	71 (47.3)	93 (93.0)	< 0.001	4.532 (2.930–7.012)
		AA	8 (5.3)	0 (0.0)	1	–
	Recessive	GG + GA Vs AA	142 (94.6)	100 (100)	–	Reference
			8 (5.3)	0 (0.0)	1	–
	Over-dominant	GG + AA Vs GA	79 (52.6)	7 (7)	–	Reference
			71 (47.3)	93 (93.0)	< 0.001	4.781 (3.112–7.345)
	Dominant	GG Vs GA + AA	71 (47.3)	7 (7.0	–	Reference
			79 (52.6)	93 (93)	< 0.001	4.240 (2.747–6.544)
	Allelic	G-Allele	213 (71.0)	107 (53.5)	–	Reference
		A-Allele	87 (29.0)	93 (46.5)	< 0.001	2.128 (1.465–3.091)

Significant values are in bold.

*P* probability, *OR* Odds ratio, *CI* Confidence intervals, *P* < 0.05 = significant.

**Fig. 2:**
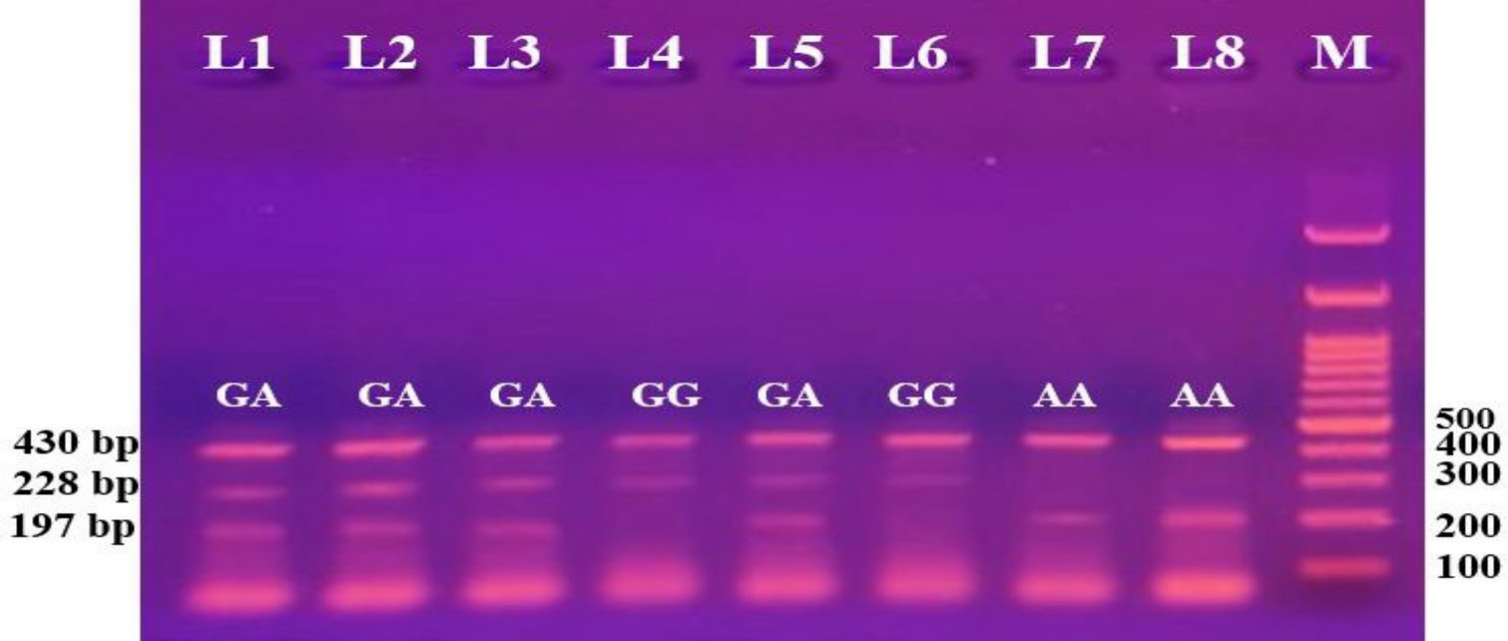
Tetra-ARMS PCR profile for the IL-10 rs1800896 product, with each lane representing one participant. M indicates a 100 bp DNA marker. The internal control is represented by the 430 bp band. According to the primer, certain 228 bp bands indicate the wild (G) allele, whereas specific 197 bp products represent the mutated (A) allele. Lanes 1, 2, 3, and 5 reflect GA heterozygosity. Lane 4 shows wild homozygous, with the A allele missing but the G allele present at 228 bp. Lanes 6 and 7 exhibit mutants’ homozygosity, revealing the A allele positioned at 197 bp while the G allele absent.

For IL-10 gene polymorphism, the heterozygous GA genotype was identified in 93% of stroke patients, higher than in controls (47.3%). Furthermore, the homozygous AA genotype was observed in healthy controls (5.3%), but not in patients. While the GG homozygous genotype was lower in patients (7%) than in controls (47.3%), the GA genotype ([OR] 4.532; (P < 0.001)), the A allele ([OR] 2.128; (P < 0.001)), the over dominant (GG + AA Vs GA) ([OR] 4.781; (P < 0.001)), and the dominant (GG Vs GA + AA) ([OR] 4.240; (P < 0.001)) genotyping model in patients illustrates a highly significant difference from volunteers that increases the risk of stroke compared to the wild type GG (Fig. 2).

### The associations between IL-1β and IL-10 genotypes and investigated parameters

The risk feature profile revealed that hypertension remained the most prevalent contributory factor among patients, occurring in 70% of instances. Followed by diabetes (52%) and smoking (46%). In addition, consciousness issues were observed in 72% of patients, and (43%) trouble chewing and swallowing.

The study investigates the association of several clinical variables with IL-1β T/C. In Table 4, the TT genotype was significantly associated with female gender (p < 0.05). A higher frequency of patients with consciousness issues was observed in the TC and CC groups compared with the TT group (p = 0.02). Regarding troubles in chewing and swallowing, a higher frequency was associated with the dominant model TC + CC group compared with the TT genotype (P = 0.03). Moreover, there was no significant link between IL-1β genetic polymorphism and DM or smoking status.

**Table 4:** Demographic, and clinical variables association with the IL-1β rs16944 polymorphisms in IS patients.

			TC n = 82	CC n = 7	TT n = 11	TC + CC n = 89	*P1*	*P2*
Gender	Male		52 (63.4%)	5(71.4%)	3 (27.3%)	57 (64.0%)	χ2 = 5.89 0.040	χ2 = 5.51 0.025
	Female		30 (36.6%)	2(28.6%)	8 (72.7%)	32 (36.0%)		
High blood pressure	Absent		24 (29.3%)	2 (28.6%)	4 (36.4%)	26 (29.2%)	χ^2^ = 0.24 *P* = 0.89	χ^2^ = 0.23 *P* = 0.62
	Present		58 (70.7%)	5(71.4%)	7 (63.6%)	63 (70.8%)		
Smoking	Absent		42 (51.2%)	3(42.9%)	9(81.8%)	45 (50.6%)	χ^2^ = 4.03 0.133	χ^2^ = 3.8 0.05
	Present		40 (48.8%)	4(57.1%)	2 (8.2%)	44 (49.4%)		
DM	Absent		40 (48.8%)	3 (42.9%)	5 (45.5%)	43 (48.3%)	χ2 = 0.12 0.123	χ2 = 0.03 0.858
	Present		42 (51.2%)	4 (57.1%)	6 (54.5%)	46 (51.7%)		
Consciousness issue	Absent		20 (24.4%)	1 (14.3%)	7 (63.6%)	21 (23.6%)	χ^2^ = 8.11 0.02	χ^2^ = 7.78 0.005
	Present		62 (75.6%)	6 (85.7%)	4 (36.4%)	68 (36.4%)		
Chewing and swallowing trouble	Absent		33 (40.2%)	2 (28.6%)	8 (72.7%)	35 (39.3%)	χ^2^ = 4.8 0.09	χ2 = 4.4 0.03
	Present		49 (59.8%)	5 (71.4%)	3 (27.3%)	54 (60.7%)		

Significant values are in bold.

*P1,* comparison between CC, TC and TT; *P2*, comparison between TC + CC vs. TT; *P* < 0.05 = significant.

Regarding IL-10 (rs1800896), the genetic dominant model (GA + AA vs. GG) was only significantly associated with troubles in chewing and swallowing among stroke patients (p = 0.018). The results show that there is no significant association between IL-10 genetic polymorphism and smoking (p = 0.337) or diabetes (p = 0.286) (Table 5).

**Table 5:** Demographic and clinical variables association with the IL-10 rs1800896 polymorphisms in IS patients.

		AA + GA n = 93	GG n = 7	Test (*P*)	p
Gender	Male	58 (62.4%)	2 (28.6%)	χ^2^ = 3.098	0.112
	Female	35 (37.6%)	5 (71.4%)		
High blood pressure	Absent	28 (30.1%)	2 (28.6%)	χ^2^ = 0.007	0.93
	Present	65 (69.9%)	5 (71.4%)		
Smoking	Absent	49 (52.7%)	5 (71.4%)	χ^2^ = 0.920	0.337
	Present	44 (47.3%)	2 (28.6%)		
DM	Absent	46 (49.5%)	2 (28.6%)	χ^2^ = 1.138	0.286
	Present	47 (50.5%)	5 (71.4%)		
Consciousness issue	Absent	25 (26.9%)	3 (42.9%)	χ^2^ = 0.82	0.36
	Present	68 (73.1%)	4 (57.1%)		
Chewing and swallowing trouble	Absent	56 (60.2%)	1 (14.3%)	χ^2^ = 5.60	0.018
	Present	37 (39.8%)	6 (85.7%)		

Significant values are in bold.

*P*_*2*_ probability, comparison between GA + AA versus GG; *U* The Mann–Whitney, T student test; *χ*^*2*^ chi-square test.

### Regression analysis for prediction of stroke incidence

Based on the provided Table 6, An analysis of logistic regression was conducted to identify predictive factors influencing stroke incidence. In the univariate analysis, several factors were examined individually to assess their association with stroke incidence. Patients with higher cholesterol, LDL, SBP, and DBP levels and lower HDL levels, together with the presence of IL-1β (rs16944) and IL-10 (rs1800896) polymorphisms, were more likely to develop stroke. However, none of the other factors reached statistical significance, including age, gender, or smoking.

**Table 6:** Regression analysis for prediction of stroke incidence.

	Univariates	
	p-value	OR (95%CI)
Age	= 0.86	1.0 (0.98–1.02)
Gender	= 0.25	0.74 (0.44–1.24)
SBP	< 0.001*	1.1 (1.07–1.14)
DBP	< 0.001*	1.11 (1.06–1.15)
Smoking	= 0.92	1.0 (0.58–1.62)
Total cholesterol	< 0.001*	1.03 (1.02–1.04)
LDL-c	< 0.001*	1.07 (1.05–1.09)
HDL-c	< 0.001*	0.69 (0.63–0.79)
IL-1β (rs16944)	= 0.028*	2.3 (1.09–4.76)
IL-10 (rs1800896)	< 0.001*	11.94 (5.19–27.45)

Significant values are in bold.

*CI* Confidence interval, *OR* Odds ratio.

## Discussion

Discovering the genetic predisposition to stroke might lead to a better understanding of the causative pathways, the development of novel treatment targets, and improved diagnostic and prognostic choices^20^. Scientists are exploring SNPs to uncover novel stroke risk factors and enhance preventative measures since they influence an individual’s genetic composition, disease risk, and treatment response, resulting in more effective therapeutic targets^21^. Numerous studies have linked cytokine levels in the blood and genetic polymorphisms to cardiovascular conditions such as myocardial infarction and stroke. The study aims to evaluate the link among the IL-1β (rs16944) and IL-10 (rs1800896) polymorphisms and ischemic stroke in Egyptians, in addition to the role of additional risk variables.

IL-1β, situated inside a 70–110 kb area of chromosome 2q13-21 comprising 7 exons plus 6 introns, is a critical mediator of the response to inflammation. It is the most well-known and researched of the 11 IL-1 members of a family, and it is necessary for the host’s immune response, including resistance to pathogens. It also worsens damage from chronic sickness and acute tissue injury^10^. IL-1β is an appropriate gene for polymorphism research owing to its relationship with inflammatory reactions. At least twenty SNPs were discovered in the IL-1β area which has been connected to the activation of febrile seizures^22^. The current investigation indicated that the genetic variation IL-1β-511T/C rs16944 was connected to stroke hazards. Homozygosity for the T and C genotypes was related to healthy people; however, the heterozygous TC genotype was more prevalent in patients than in control subjects. Over dominant TT + CC vs. TC as well as dominant models TC + CC vs. TT were more inclined to suffer stroke than the healthy group of people, but recessive models TC + TT vs. CC were less common among stroke groups than the control group, showing a protective effect against stroke. Our work supports prior findings linking the IL-1β promoter polymorphism at the -511-T/C location to IS risk and related disorders^23^. The same results were shown in myocardial infarction and ischemic stroke in young patients^24^. However, a prior study in Tunisia found that the IL-1β gene polymorphism might not be a risk contributor to ischemic stroke^9^. Thus, while the C-allele is not directly connected to IS, it may affect cytokines in inflammatory reactions that influence the course of atherosclerosis, eventually leading to IS.

IL-10, a cytokine generated by monocytes and lymphocytes, is a crucial anti-inflammatory cytokine that can rise in plasma, cerebral fluid, and blood mononuclear cells in individuals with acute stroke^25^. It balances out the negative effects of TNF along with additional pro-inflammatory chemicals^12^. However, earlier research has produced mixed outcomes. A Brazilian study showed no relationship between IL-10 haplotypes and ischemic stroke risk in sickle cell anemia children^26^. A meta-analysis investigated that the IL-10 (A/G) rs1800896 mutation is substantially related to individual vulnerability to IS in the recessive model, AA versus GG + GA (p = 0.001, OR = 1.42) in the overall population^15,27^. Previous results revealed that both homozygotes AA and heterozygotes AG genotypes of IL-10–1028 A/G were more frequent in stroke patients than healthy individuals, and the A allele was thought to be a risk contributor to IS in the Turkish population^28^. Consistent with all these studies, the study indicated that stroke patients had a greater prevalence of the heterozygous carrying (GA) genotype, bearing a ~ fourfold risk of getting a stroke. This risk was considerably higher under multiple genetic models, including the dominant, over-dominant, and allelic models.

Modifiable risks such as hypertension, smoking, and high lipid levels all contribute significantly to stroke incidence, and knowing these risk factors is critical for stroke prevention^29^. In physical manifestation, 46% of individuals smoked, increasing their risk of stroke. Smokers are twice as likely to experience a stroke and account for 15% of all stroke-related fatalities^30,31^. While the global incidence of smoking within stroke patients is substantial, in this study, cigarette smoking did not show up to be a significant danger factor according to a regression analysis. This might be due to the reality that the study encompassed female patients, a category in Egyptian society in which smoking is usually low. Therefore, this might have undervalued the impact of smoking as a risk aspect in the study results. The study found that 70% of patients had greater systolic and diastolic blood pressure than controls (P < 0.001). Most studies indicate that elevated SBP is associated with a higher risk of strokes and heart disease than elevated DBP, with 54% of stroke victims having both SBP and DBP^32^. Hypertension causes stroke through a variety of processes, including endothelial alterations and damage, increased blood–brain barrier permeability, and altered blood-endothelial interactions. These modifications encourage the development of thrombi, ischemic lesions, fibrinoid necrosis, and lacunar infarctions. Additionally, hypertension accelerates arteriosclerotic processes, which increases the chance of cerebral lesions^33^.Diabetes considerably raises the risk of lacunar strokes, doubles the likelihood of ischemic stroke (IS), and is linked to a 20% higher fatality rate. Diabetic people typically have a worse prognosis after a stroke, with severe impairment and a delayed recovery^34^. According to research, 52% of people with IS had diabetes, with diabetics having much higher glucose levels than non-diabetics. Furthermore, thrombi in diabetic individuals are more resistant to treatment because of their distinct fibrin and erythrocyte composition^35^. Hyperlipidemia is a major precursor for blood vessel atherosclerosis and strokes. The current study discovered that patients with stroke had significantly greater TC, LDL-C, and low HDL-C levels than the control group, which is similar to recent research in India that reported lower HDL-c values in stroke patients^22^. The inverse relationship between HDL-C and stroke may be due to HDL-C's ability to transport cholesterol and prevent lipid peroxidation^36^. Stroke patients showed significantly lower serum triglycerides compared to the control group, which is consistent with previous studies^37^, contradicting a study in India that found no significant difference in triglyceride levels^38^.

No significant correlation was found between age at illness onset or gender and the A1082G polymorphism. Also, no significant correlation was found with blood pressure. However, the genetic dominant model (GA + AA) is substantially related to a greater probability of individuals experiencing chewing and swallowing issues than the GG genotype. We can compare the results of this study with a preceding one that found a significant association between the rs1800896 (A1082G) SNP and stroke due to ischemic attack in a South Indian populace in genotypic dispersion and allelic frequency (P < 0.05)^12^. The SNP was also associated with strokes of undetermined etiology.

In conclusion, for the rs16944 (T/C) and rs1800896 (A/G) polymorphisms, prior case–control research overall analysis revealed significant heterogeneity, implying that the genetic distribution of these polymorphisms differs substantially between groups, providing a more definite outcome for the Egyptian population and IS. One of the major obstacles in the present study is the limited sample size; while the sample size is acceptable for a preliminary study, larger multicenter studies should be encouraged for validation and increased statistical power as well as the presence of interfering conditions like diabetes. Using a larger number of patients to study mutations will aid in the diagnosis and early detection of stroke patients; while ruling out some interfering factors is a promising requirement.

## Data Availability

Data is provided within the manuscript all data in the manuscript.
